# Golgi dysfunction in Alzheimer disease: from human multiomic signatures to therapeutic targets

**DOI:** 10.1038/s12276-026-01851-8

**Published:** 2026-10-02

**Authors:** Jaehoon Song, Seung-Jae Lee, Inhee Mook-Jung

**Affiliations:** 1 Department of Biomedical Sciences, Seoul National University College of Medicine, Seoul, Republic of Korea; 2 Convergence Dementia Research Center, Medical Research Center, Seoul National University, Seoul, Republic of Korea

**Keywords:** Cellular neuroscience, Alzheimer's disease

## Abstract

Deciphering the intricate pathogenesis of Alzheimer disease (AD) has been a highly demanding task for researchers for decades. Evidence from from human multiomic studies provides critical insights into complex disease mechanisms and drives novel findings. However, the data are often selectively comprehended, leaving a few notable factors underappreciated in the research field. We focus on the Golgi apparatus, one of the most undervalued organelles in AD research, to demonstrate its multidimensional contribution to the pathogenesis. Given the importance of Golgi in modulating intracellular protein and lipid homeostasis, implication of Golgi in AD pathogenesis deserves thorough investigation. In this review, we describe changes in Golgi morphology and function in AD, and summarize specific Golgi-related factors discovered by genomic, transcriptomic, proteomic, and lipidomic data using human samples. Based on the findings, we demonstrate mechanistic link of Golgi-related factors to AD pathogenesis and highlight potential therapeutic strategies to modify Golgi-mediated pathogenesis. Overall, Golgi dysfunction may serve as a notable mechanistic hub of AD pathogenesis, making it a promising target for development of novel therapeutic strategies.

## Golgi dysfunction revealed as a key player in Alzheimer disease

Alzheimer disease (AD) is a leading cause of cognitive decline in the elderly, characterized by amyloid plaques and neurofibrillary tangles. Recent studies highlight the Golgi apparatus, a key organelle in protein and lipid processing, as a notable player in AD pathogenesis. This review explores how Golgi dysfunction may contribute to AD by affecting amyloid precursor protein (APP) processing and lysosomal function, based on evidence from multiomic studies. The authors discuss genetic risk factors linked to Golgi-related genes, such as *SLC9A7* and *SORL1*, which influence APP trafficking and amyloid-beta production. Findings suggest that Golgi dysfunction could exacerbate amyloid pathology and synaptic degeneration through disrupted protein modifications and lipid homeostasis. The review proposes targeting Golgi integrity and function as a potential therapeutic strategy, emphasizing the need for further research to validate these approaches and explore their clinical applicability in AD treatment.

This summary was initially drafted using artificial intelligence, then revised and fact-checked by the author.

## Introduction

Alzheimer disease (AD) is a memory-affecting disease characterized by the accumulation of amyloid plaques and neurofibrillary tangles in the brain, which leads to the loss of neurons and ultimately cognitive decline^1^. It is not only the most prevalent neurodegenerative disease but also one of the major causes of death in the elderly. According to the 2024 data, an estimated 10.9% of Americans aged 65 and older had AD, and it was the fifth leading cause of death in that population^2^. Given this clinical importance, numerous researchers worldwide have struggled for decades to elucidate the cause and pathophysiology of this disease. Owing to such efforts, a great deal of findings has revealed important aspects of disease pathophysiology; however, critical knowledge gaps remain to be addressed.

Anti-amyloid antibodies, lecanemab and donanemab, have been approved as new drugs for AD since 2023. These antibodies promote clearance of amyloid plaques and significantly delay cognitive decline^3,4^. Proof of concept that removing toxic amyloid plaques can lead to improved clinical outcomes supports the old ‘amyloid cascade theory’^5^. However, the limited effectiveness of anti-amyloid antibodies emphasizes the demand for therapeutic strategies targeting other aspects of the disease. One of the most promising strategies is to resolve predisposing conditions that cause the production of toxic amyloid-β (Aβ) species, as this method would have a complementary and synergistic effect with the amyloid plaque-clearing antibodies. Aβ is produced by the consecutive cleavage of amyloid precursor protein (APP) by β-secretase and then by γ-secretase^6^. Numerous studies have suggested factors that increase Aβ production or alter the ratio of Aβ isoforms. However, drugs targeting those mechanisms (e.g., β-secretase inhibitors) have failed to show clinical benefit until now, indicating an urgent need for elucidating most critical, earliest pathogenic mechanism of AD.

In sporadic AD, which accounts for approximately 90% of total AD, various risk factors contribute to development and progression of the disease, including genetic factors, environmental factors, and aging^7^. This multidimensional nature of the disease makes it extremely difficult to decipher exact pathogenesis. Although recent multiomic studies have revealed important clues, our current knowledge lets us interpret only part of the data. As clinical and experimental data in AD research accumulates rapidly, researchers face increasing need to weigh the importance and quality of evidence alongside its relevance to actual human disease. However, some aspects that have the potential to fill the knowledge gap of the field are often undervalued.

In this review, we address recent discoveries and possible pathogenic mechanisms regarding one of the most underrepresented organelles in the field of AD research: Golgi apparatus. Evidence from human data is mainly addressed, including data from genetic studies and multiomic studies with AD brain samples. We discuss possible contribution of Golgi to pathogenic mechanisms of AD and how the risk factors of AD relate to those Golgi-related mechanisms. Moreover, we propose potential therapeutic strategies targeting Golgi dysfunction in AD, which may represent a paradigm shift in positioning Golgi as a causal node in AD pathogenesis.

## Golgi in health and AD

### Physiologic role of the Golgi apparatus

The Golgi apparatus is a subcellular organelle that is referred to as the ‘post office of a cell’. Its main role is sorting and processing protein cargos produced in the endoplasmic reticulum (ER)^8^. It also receives protein cargos from other sites, such as the plasma membrane and endosomes. In Golgi, proteins are thoroughly sorted and processed through protein modification, including glycosylation and proteolytic processing^8^. Regulation of protein sorting to accurate organelles is critical to maintaining normal cell physiology. For example, mannose 6-phosphate (M6P) modification of lysosomal proteases in Golgi is a major signal for trafficking to lysosomes^9^.

Besides its well-known role, Golgi plays various roles in protein quality control. Golgi proteins are involved in autophagosome formation, maturation, and fusion with lysosomes^10–12^. In addition, Golgi has its intrinsic protein degradation pathways, such as the proteasome-dependent endosome and Golgi-associated degradation and the autophagy-resembling Golgi membrane-associated degradation^13^.

Golgi is also involved in lipid metabolism. Various glycolipids are generated by glycosylation in Golgi, including sphingomyelin and glycosphingolipids—major components ōf the plasma membrane^14^. Furthermore, several intracellular signaling pathways occur at Golgi to regulate not only its morphology and function, but also energy metabolism, cytoskeleton organization, and cell death^15,16^. Collectively, Golgi plays diverse roles in regulating dynamic cellular homeostasis.

### Morphological and functional changes of Golgi in AD

Morphological changes of Golgi, including Golgi fragmentation, were observed in the postmortem brains of patients with AD^17–19^. Furthermore, Golgi fragmentation was induced by the expression of APP Swedish mutant (APPswe), and the recovery of Golgi structure via expression of Golgi structural proteins GRASP65 and GRASP55 (Golgi Reassembly Stacking Protein 65/55) drastically reduced Aβ production, suggesting its relevance in AD pathogenesis^20^. Fragmented Golgi phenotype was preserved in induced pluripotent stem cell-derived neurons from patients with sporadic AD^21^. Interestingly, fragmentation preceded the development of amyloid and tau pathology in these cells, indicating that Golgi fragmentation may be an early pathogenic change in AD progression.

Dysregulation of Golgi morphology and function has been linked to other pathologic hallmarks of AD. Elevated intracellular phosphorylated tau levels correlate strongly with the reduction of Golgi volume in the brains of patients with AD^19^. In physiologic settings, tau mediates the interaction between Golgi membrane and microtubules^22^. Interestingly, two studies have reported that that pharmacological induction of Golgi fragmentation increases tau phosphorylation via activation of GSK-3β and Cdk5, the primary mediators of tau hyperphosphorylation in AD^23,24^. Furthermore, neuronal hyperexcitability can induce Golgi fragmentation^25^, a process that has been suggested to increase tau secretion from neurons and accelerate the spread of pathologic tau seeds in AD^26^. These findings collectively led to generation of hypothesis that Golgi fragmentation contributes to a wide range of AD-related pathogenic changes^27^.

## Recent findings revealing link between Golgi and AD from human data

### Golgi-related genetic risk factors of AD

Recent analysis of a large RNA-sequencing dataset comparing the polygenic risk score of AD, which quantifies the genetic risk of an individual, with the actual disease morbidity revealed that Golgi-related gene ontology terms were elevated in both patients with AD and people with high polygenic risk score of AD^28^. This elevation was more significant in the temporal cortex than in the cerebellum, further supporting specificity in AD pathogenesis. Suggested as an underrepresented disease-related organelle, Golgi holds substantial potential for involvement in AD pathogenesis.

Several studies have revealed gene loci related to Golgi function in patients with AD. In an X-chromosome-wide association study, *SLC9A7* (solute carrier family 9 member A7) has been reported as the only genome-wide significant risk locus^29^. SLC9A7, also known as NHE7 (sodium-hydrogen exchanger 7), is a cation (Na^+^, K^+^)/proton (H^+^) exchanger that resides in the Golgi, with preferential accumulation in the *trans*-Golgi network (TGN) and post-Golgi vesicles^30^. SLC9A7 plays a role in maintaining the pH homeostasis and function of the TGN^31^. Because alterations in Golgi luminal pH can dysregulate protein modification, glycosylation, trafficking, and recycling^32–34^, the disruption of SLC9A7 function would affect various protein metabolisms, including APP processing.

*SORL1* (sortilin-related receptor 1) is a well-known strong genetic risk factor of AD identified from genome-wide association studies (GWAS)^35^. SORL1 (SORLA) is an endosomal receptor that mainly mediates trafficking to TGN. Previous reports have shown that SORL1 directly interacts with APP and regulates its trafficking to TGN, reducing amyloidogenic cleavage at endosomes^35–37^. *SORCS1* (sortilin-related VPS10 domain containing receptor 1), also a member of sortilin family, is also a recognized genetic risk factor for AD^38^. Previous studies have reported that reduced SORCS1 in AD can decrease APP localization to TGN, thereby increasing γ-secretase cleavage and subsequent Aβ production^39,40^.

Meanwhile, *AP4M1* and *AP4E1*, encoding components of adaptor protein 4 complex, have been annotated as risk genes by enhancer mapping of AD GWAS risk loci^41^. Increased AP4M1 expression was predicted to be associated with increased AD risk. AP4M1 (the μ4 subunit) reportedly interacts with APP and regulates its delivery to endosomes^42^. Therefore, increased AP4M1 expression may also lead to increased Aβ production at endosomes.

A genetic variant of the *NSF* (N-ethylmaleimide sensitive factor) gene was reported as one of four significant AD-related variants in whole-exome sequencing analysis of non-ApoE4 patients with AD^43^. This variant was correlated with reduced risk of AD. NSF is involved in the regulation of Golgi reassembly and vesicle fusion at Golgi^44,45^. The missense mutation of *NSF* resulted in smaller Golgi size and occurrence of abnormal cisternae, highlighting its importance in Golgi integrity^46^. Although the exact functional implication of NSF genetic variant in AD pathogenesis remains unknown, the findings point out that maintenance of Golgi integrity can provide resilience to AD pathogenesis.

In a 2025 study, machine learning analysis of European GWAS has suggested *COG7* (component of oligomeric Golgi complex 7) as a new risk gene of AD^47^. COG7 consists of COG complex, which regulates Golgi functional and morphological homeostasis. The mutation of this protein causes glycosylation defects^48^, implying that dysfunctional COG7 activity can lead to aberrant glycosylation of various proteins as observed in the brains of patients with AD^49,50^. COG7 was also implicated as an AD causal protein in the protein quantitative trait loci (pQTL) analysis of multiancestry brain proteomic data, further supporting the strength of evidence^51^. Interestingly, several Golgi-related proteins were also nominated as causal proteins of AD in the study, including the TGN-resident metalloprotease CPD (carboxypeptidase D)^52^ and proteins involved in endosome-to-Golgi retrograde trafficking (SNX1, SNX32, STX6)^53^.

A hippocampal transcriptome-wide association study, which evaluated genetically regulated gene expression associated with AD, has identified *QPCTL* (glutaminyl-peptide cyclotransferase-like protein) as the top AD risk gene^54^. In this study, *QPCTL* expression was positively correlated with hippocampal volume and negatively correlated with AD risk. QPCTL is a Golgi-localized pyroglutamation enzyme that converts glutamine or glutamate residue into pyroglutamate^55^. QPCTL modifies CD47 to enhance its interaction with SIRPα^56^, a process that protects neuronal synapses from phagocytosis^57^. Therefore, impaired QPCTL function in Golgi would contribute to excessive synaptic engulfment, possibly causing neurodegeneration in AD. However, the link between QPCTL and AD needs further validation, as a previous study has reported contradictory results showing increased QPCTL expression in the brains of patients with AD^58^.

**Fig. 1:**
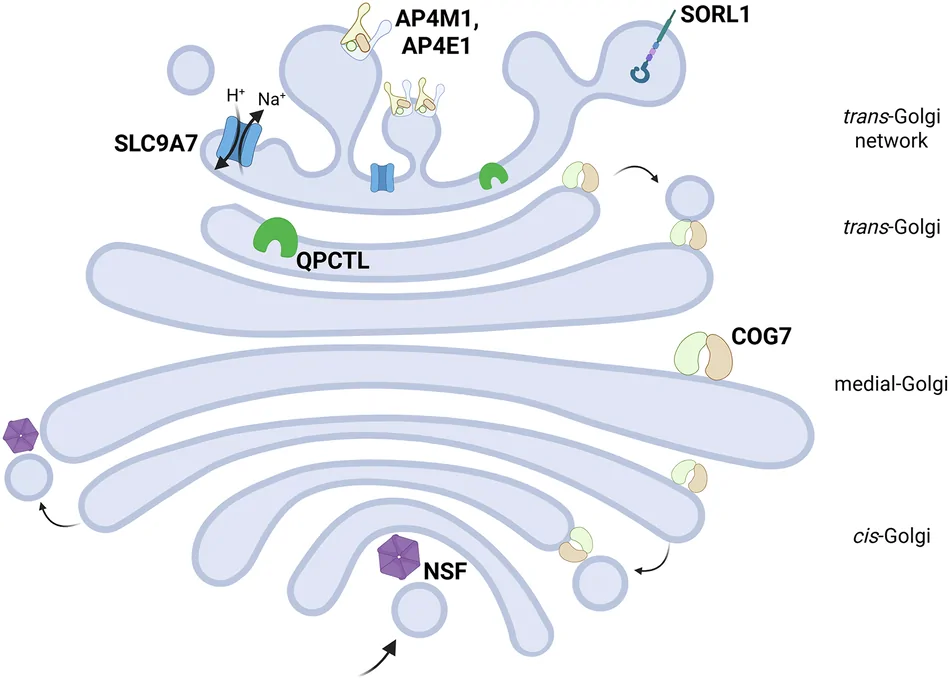
Localization of Golgi-related genetic risk factors in AD. Depicted subcompartmental localizations are approximate and may vary with cell type and physiologic conditions; the schematic is intended to convey functional relationships rather than fixed positions.

The abovementioned genetic risk factors collectively imply the potential involvement of Golgi in AD pathogenesis. Moreover, considering that majority of those risk factors localize at TGN (Fig. 1), disruption of processes in TGN may be the critical pathogenic contributor.

### Trafficking and sorting defects in AD

Golgi is a recognized regulator of protein trafficking and sorting. It not only sends cargos to other organelles, but also receives cargos from endosomes or plasma membrane via retrograde trafficking. Multiple genetic risk factors of AD, including SORL1, SORCS1, SNX1, SNX32, and STX6, strongly support the pathogenic implication of retrograde trafficking from endosome to TGN, considering that endosome is considered as the primary site of amyloidogenic cleavage^59^. As described in the previous section, experimental evidence suggests that APP trafficking back to the Golgi is critical to maintaining the physiologic level of Aβ production. Disruption of this process results in APP accumulation in endosomes, predisposing APP to amyloidogenic cleavage.

**Fig. 2:**
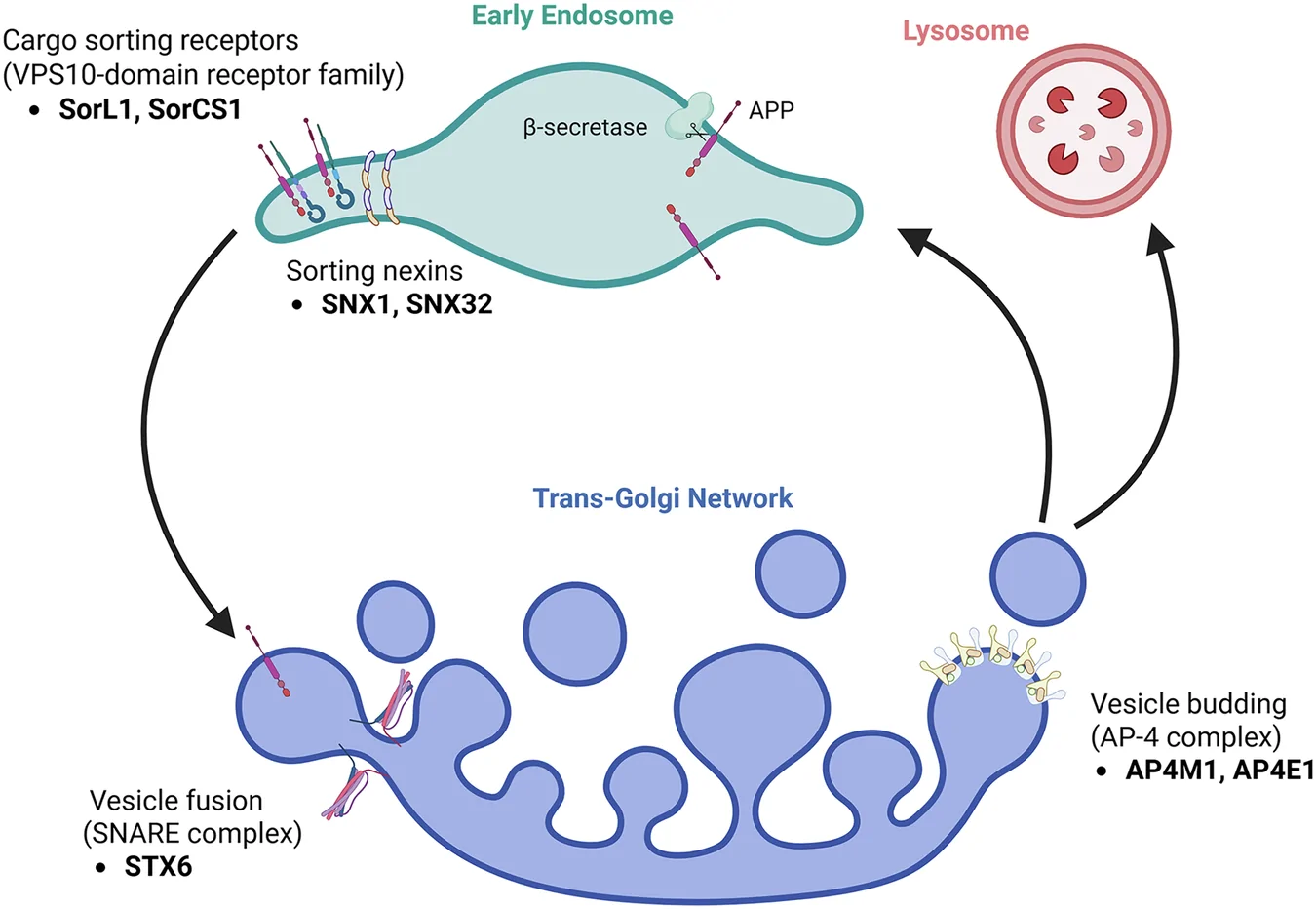
Golgi trafficking-related AD risk factors.

On the contrary, trafficking between Golgi and lysosomes is also critical to Aβ pathology. Lysosomal dysfunction is one of the earliest phenotype in AD^59^. Decreased degradation in lysosomes leads to abnormal accumulation of Aβ, along with other undegraded protein aggregates. Aβ further compromises lysosomal function, forming pathogenic vicious cycle^59^. Notably, Golgi regulates anterograde trafficking of various proteins to lysosomes, including essential lysosomal proteases, and receives retrograde cargos from lysosomes^60^. Defects in Golgi can impair lysosomal biogenesis and function, mainly via mis-sorting of lysosomal proteins^61,62^. Interestingly, post-Golgi carriers convey lysosomal enzymes to axonal endosomes and autophagic vacuoles, and these carriers accumulate in dystrophic neurites in AD^63^. This result suggests that trafficking of lysosomal enzymes from Golgi is specifically essential to degradation of endosomal contents in axons. Because adaptor protein complex 4 is involved in vesicle budding from TGN and regulates lysosome function^64^, AD risk loci affecting *AP4M1* or *AP4E1* expression may be related to altered lysosomal function^41^. Taken together, Golgi dysfunction can promote amyloid pathology by reducing APP retrieval from endosomes and impairing lysosomal degradation (Fig. 2). Restoring these trafficking processes, particularly the retrograde trafficking of APP, may represent a promising therapeutic strategy for AD.

### Glycosylation and matrisome dysregulation in AD

Proteins undergo various modification processes after being translated, generating a large gap between the transcriptome and the actual proteome. A large-scale proteomic study conducted in 2022 analyzed more than 500 dorsolateral prefrontal cortex tissues from patients with AD and controls via tandem mass tag–mass spectrometry-based quantitative proteomics^65^. In this study, ‘Golgi’ and ‘protein transport’ module eigenproteins were decreased in the brains of patients with asymptomatic AD and further decreased in those with symptomatic AD. These module eigenproteins showed positive correlation with MMSE (Mini-Mental Status Examination) scores and global cognition, indicating that the loss of Golgi-related proteins is associated with cognitive decline in AD.

**Fig. 3:**
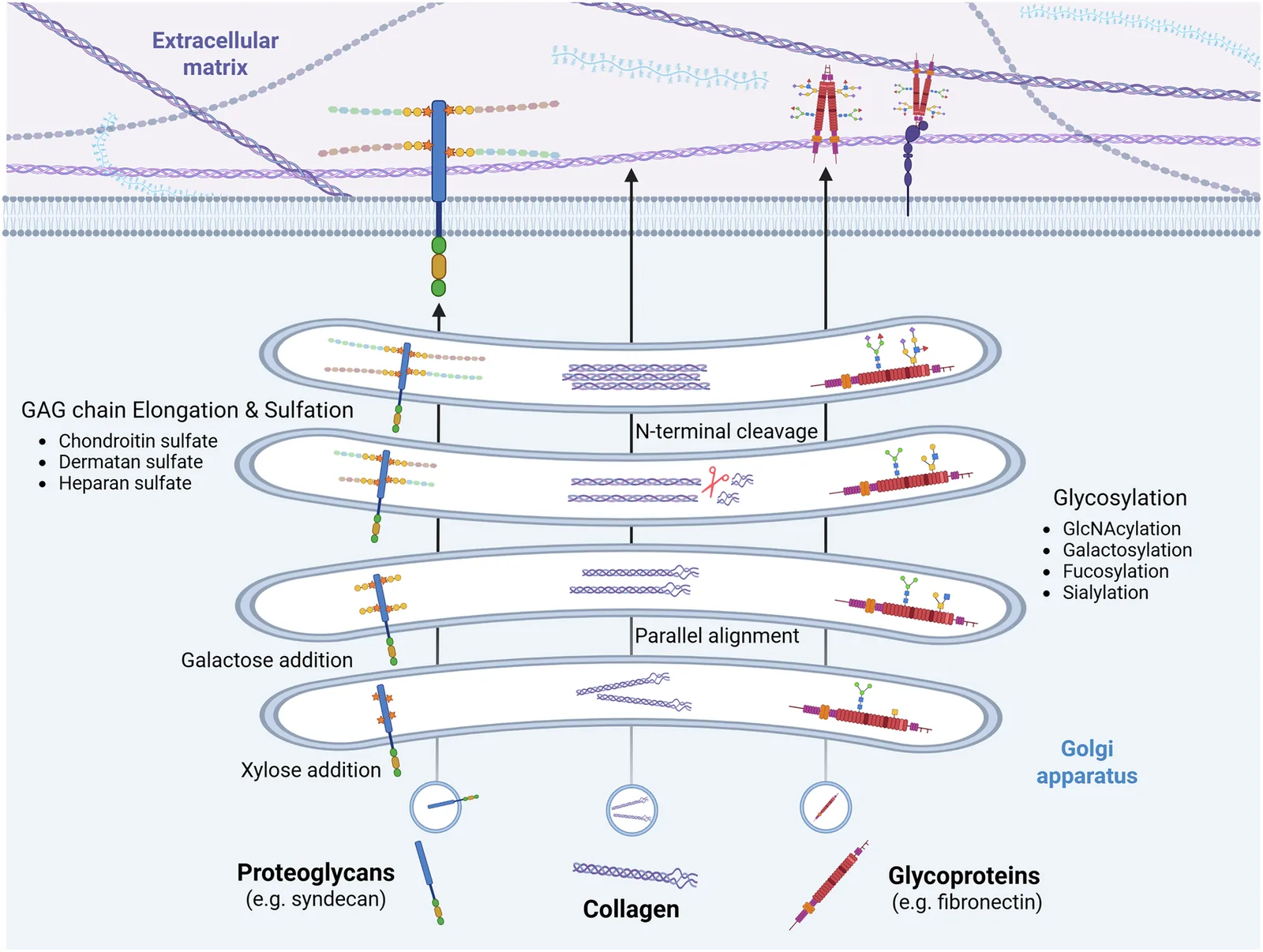
Matrisome protein processing in Golgi.

The matrisome, a group of proteins comprising the extracellular matrix (ECM), represented the most prominent proteomic signature in AD that is not well presented in the transcriptome^65,66^. This discordance indicates that the critical changes of ECM proteins in AD are mainly due to changes in posttranslational modifications. Because ECM proteins are largely modified in the Golgi before being secreted to the extracellular space, Golgi dysfunction can lead to disruption of ECM environment^67^ (Fig. 3).

Proteoglycans, the core components of ECM, are mainly processed in the Golgi, where glycosaminoglycan (GAG) chains are added to the O-xylose of proteoglycans^68^. Various Golgi-resident enzymes, including xylosyltransferases, galactosyltransferases, glucuronyltransferases, and sulfotransferases, contribute to proper modification of proteoglycans^67,69^. Collagen, another major element of ECM, undergoes N-terminal cleavage and parallel alignment in Golgi^67,70^. Indeed, the knockout of specific golgins resulted in impaired collagen fibril secretion and ECM organization^71^. Other glycoproteins of the ECM, including fibronectin, are also glycosylated in Golgi^72^. The importance of matrisome processing in Golgi may hold clues to elucidate critical pathogenic mechanisms in AD. Significant alterations in the matrisome that are not exhibited in transcriptomic studies underscore the need to investige Golgi-mediated pathogenic protein processing mechanism. Notably, matrisome proteins colocalize with amyloid plaques, suggesting involvement in amyloid pathology^66^. Matrisome glycosylation is also involved in neuroinflammation via various mechanisms, but specific role of Golgi-dependent modification remains unclear^73^. Whether Golgi dysfunction-induced changes in matrisome glycosylation drive AD pathogenesis remains to be elucidated.

Beyond matrisome, protein glycosylation is implicated in a wide range of AD-related biological processes, including neuroinflammation, neuronal function, and synaptic plasticity^74,75^. A 2024 glycomics study has revealed changes in the N-glycan landscape in the brains of patients with AD^76^. The most remarkable differences between the AD and control group were observed in sialylated and highly branched forms, whose modifications occur within the Golgi apparatus. Components of plasma membrane, extracellular space, and synapse that were involved mainly in cell adhesion and cell communication were the most affected glycosylated proteins in AD. Particularly, the most substantially altered glycoforms were directly related to synaptic function. Glycoform module 1, which was positively correlated with disease status, comprised the glycosylation of L1CAM (L1 cell adhesion molecule) and NPTX1 (neuronal pentraxin 1). L1CAM is an adhesion molecule involved in synaptic plasticity and has been reported to reduce amyloid pathology^77^. NPTX1 is a synaptic protein that has recently been identified as a marker of synaptic loss in AD^78^. Conversely, glycoform module 3, which was negatively correlated with disease status, mainly comprised the glycosylation of ATP1B1 (sodium/potassium-transporting ATPase subunit beta 1) glycosylation. ATP1B1 is a subunit of the sodium/potassium pump that generates membrane potential for neural signaling and synaptic transmission and has been linked to AD in a previous proteomic study^79^. Furthermore, the expression of several Golgi-resident glycosylation enzymes (MAN2A1, MGAT1, B3GALT2, B4GALT1, ST6GAL2, ST8SIA2, and ST8SIA3) was altered in multiple brain regions of patients with AD^76^. These results support the claim that the glycosylation process within Golgi is altered in AD and may play a critical role in pathogenesis, including synaptic degeneration.

### Lipid dyshomeostasis and organelle crosstalk

Intracellular lipid accumulation is another key pathological feature of AD described by Alois Alzheimer^80^. Various genetic risk factors of AD have been linked to lipid processing and lipid droplet formation, including the famous risk factor *APOE*^81^. Numerous studies have reported lipidomic changes in the AD brain, including elevated levels of ceramide, cholesterol, and cholesterol esters, alongside a reduction in phospholipids^82,83^.

Ceramide is a form of sphingolipid that contributes to a wide range of cellular signaling processes^84^. Elevated ceramide levels are directly involved in increased Aβ production, oxidative stress, and neuronal apoptosis^85^. Golgi is the primary site of ceramide processing, where glucosylceramide is produced by glucosylceramide synthase (GCS, also known as UGCG) residing in *cis*-/*medial*-Golgi to be further processed into glycosphingolipids^86^. GCS levels are reportedly decreased in AD, and this reduction is correlated with ceramide accumulation^87^. Pharmacological inhibition of GCS in neurons leads to increased long-chain ceramides and neurite swelling, which resembles dystrophic neurites in AD^87^. Reduced GCS activity at Golgi may be one of the causal factors of ceramide accumulation observed in AD. Moreover, neutral sphingomyelinase 2 (nSMase2, also known as SMPD3), a ceramide-producing enzyme mainly localized in Golgi and plasma membrane, is involved in the formation of secretory extracellular vesicles, which can transmit pathologic molecules between cells, including pathologic tau proteins^88,89^. Indeed, pharmacological inhibition of neutral sphingomyelinase 2 decreased tau propagation and neurodegeneration in a tau mutant mouse model^90^. Another study has reported that neuronal *SMPD3* knockout reduced Aβ production^91^. Despite mechanistic evidence, nSMase2 activity in patients with AD remains to be validated, as a slight reduction in *SMPD3* expression in the temporal cortex of patients with AD has been reported in transcriptomic studies^92^.

Cholesterol is a critical component of cellular membranes that regulates the stability and fluidity of the membrane and is also critical to the formation of localized lipid ‘rafts’ of membrane^93^. Notably, lipid rafts are first assembled in Golgi^94^. In addition, protein trafficking throughout Golgi depends on cholesterol balance of the membrane; thus, maintenance of optimal cholesterol level is critical to Golgi function^95^. Elaborate regulation of cholesterol level is particularly important, as previous studies have shown that an increase in the membrane cholesterol affects amyloidogenic processing of APP in lipid rafts to increase Aβ production, along with reduction in Aβ clearance and induction of several cellular phenotypes seen in early AD^96–98^.

Although the Golgi function is affected by cholesterol, Golgi also actively regulates the cholesterol gradient between membranous organelles, high in the plasma membrane and low in the ER, via direct membrane contact between TGN and ER^99,100^. Oxysterol-binding protein 1 (OSBP1) is a crucial protein that regulates Golgi PI4P–ER cholesterol exchange at their contact sites^101^. A reduction in OSBP1 activity can cause cholesterol accumulation in the ER and a subsequent increase in cholesterol esters and lipid droplets^102^, whereas PI4P accumulation in Golgi can cause excessive PI4P transfer to mitochondria, resulting in abnormal mitochondrial fission^103^. Therefore, OSBP1 dysfunction may cause mitochondrial defects, which occur even before plaque accumulation in AD and affect cellular energy metabolism, oxidative stress, plaque formation, and neurofibrillary tangle formation^104^. Moreover, the inhibition of OSBP1 results in partial degradation of Golgi and reduced retrograde trafficking^101^. Notably, OSBP1 induces APP localization to Golgi, and knockdown of *OSBP* increased the amyloidogenic processing of APP^105^. This study suggests a causal relationship between Golgi-regulated cholesterol homeostasis and amyloid pathology.

**Fig. 4:**
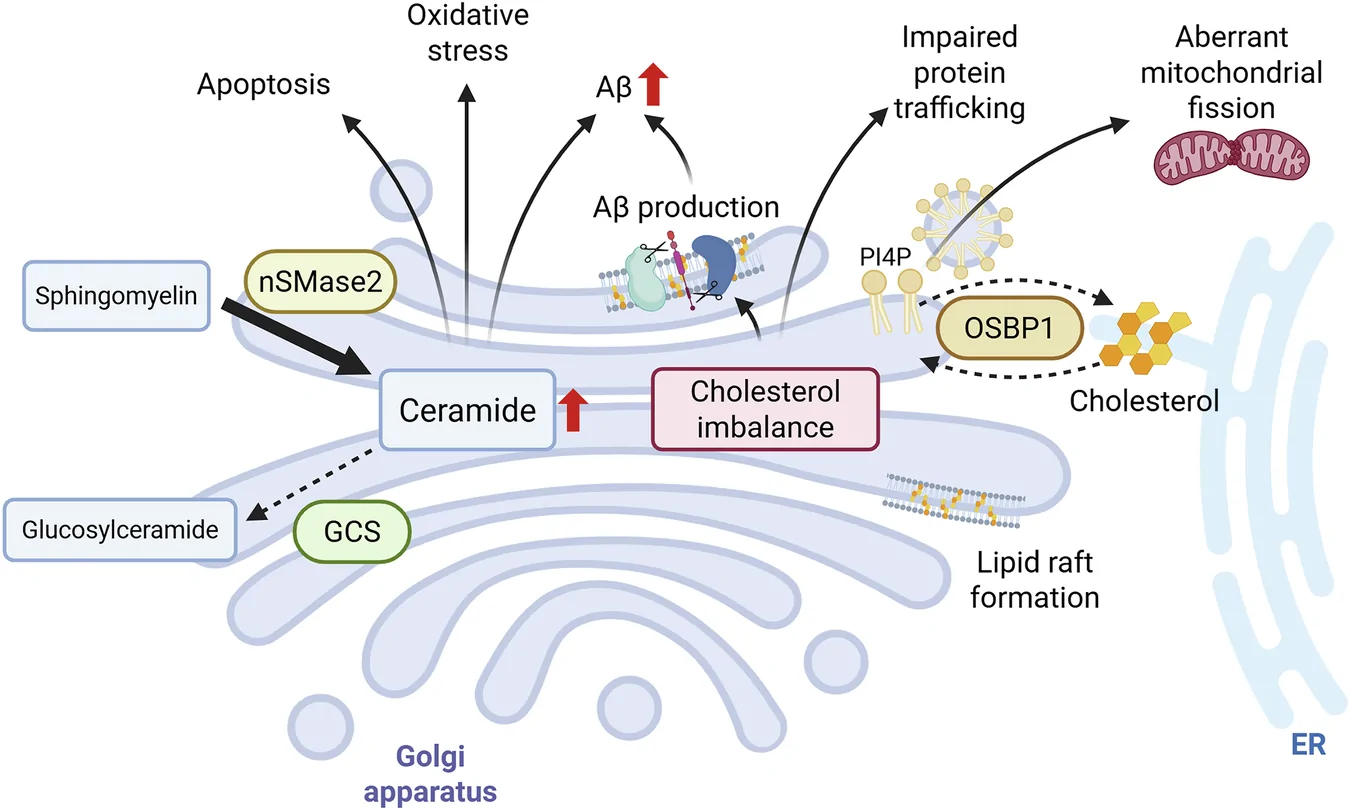
Golgi-related lipidomic changes and organelle crosstalk in AD.

Notably, direct TGN–ER contact also contributes to regulating protein secretion. Sorting and export of secretory cargos at TGN are dependent on Ca^2+^ influx into TGN^106^. A recent study has demonstrated that IP3 receptor-mediated Ca^2+^ efflux from ER provides high local Ca^2+^ concentration at TGN–ER contact sites to facilitate Ca^2+^-dependent protein secretion, and disruption of TGN–ER contact can impair this process^107^. Compromised structural and functional integrity of Golgi may impair the regulation of lipid homeostasis and protein secretion, potentially promoting pathologic changes in AD (Fig. 4). Furthermore, although pharmacological intervention of lipids can have limited effects and cause side effects because of the complexity of metabolic pathways and intracellular distribution, direct modulation of Golgi enzymes such as GCS, nSMase2, or OSBP1 can be an alternative therapeutic option to restore intracellular lipid homeostasis. Collectively, Golgi deserves more attention in studying lipid-mediated pathogenic mechanisms in AD, considering its role in modulating intracellular lipid homeostasis.

## Possible pathogenic mechanisms

**Fig. 5:**
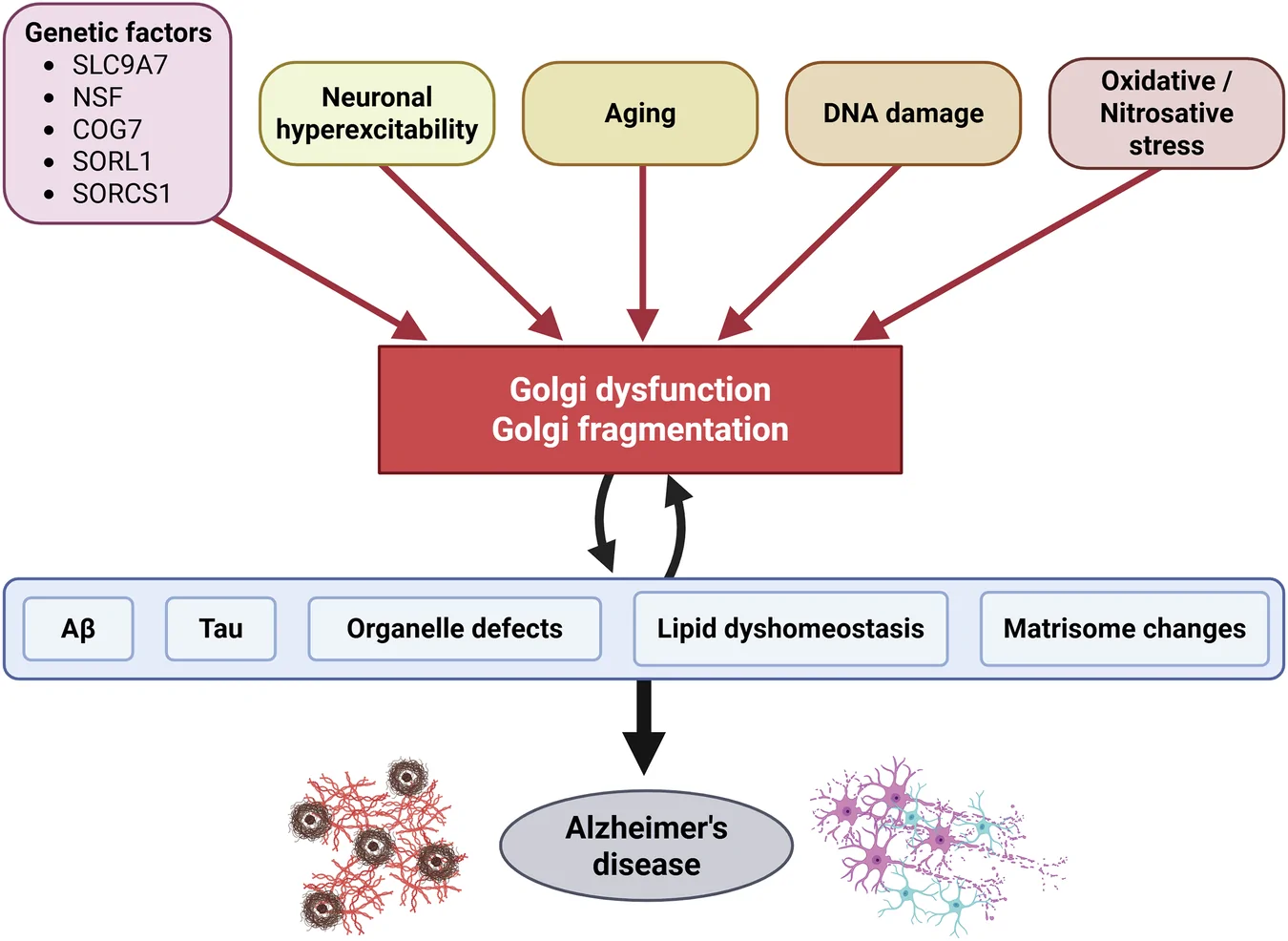
Central hub hypothesis of Golgi dysfunction in AD.

Various factors related to AD have been studied to induce changes in Golgi morphology and function (Fig. 5). As mentioned above, neuronal hyperexcitability causes fragmentation of Golgi^25^. Neuronal hyperexcitability is related to cognitive decline in patients with AD^108^. Aging, the most prominent risk factor of AD, has been reported to alter Golgi morphology and increase Golgi stress^109,110^. DNA damage, which is also closely related to aging, induces Golgi fragmentation via DNA damage protein kinase-mediated phosphorylation of Golgi phosphoprotein 3 (GOLPH3)^111^. Oxidative and nitrosative stress are other causes of Golgi fragmentation^112^, and oxidative stress impairs both anterograde and retrograde trafficking of proteins by specifically disrupting the TGN^113^. Last but not least, Aβ and tau can induce Golgi fragmentation, leading to a vicious pathogenic cycle^20,114^.

**Fig. 6:**
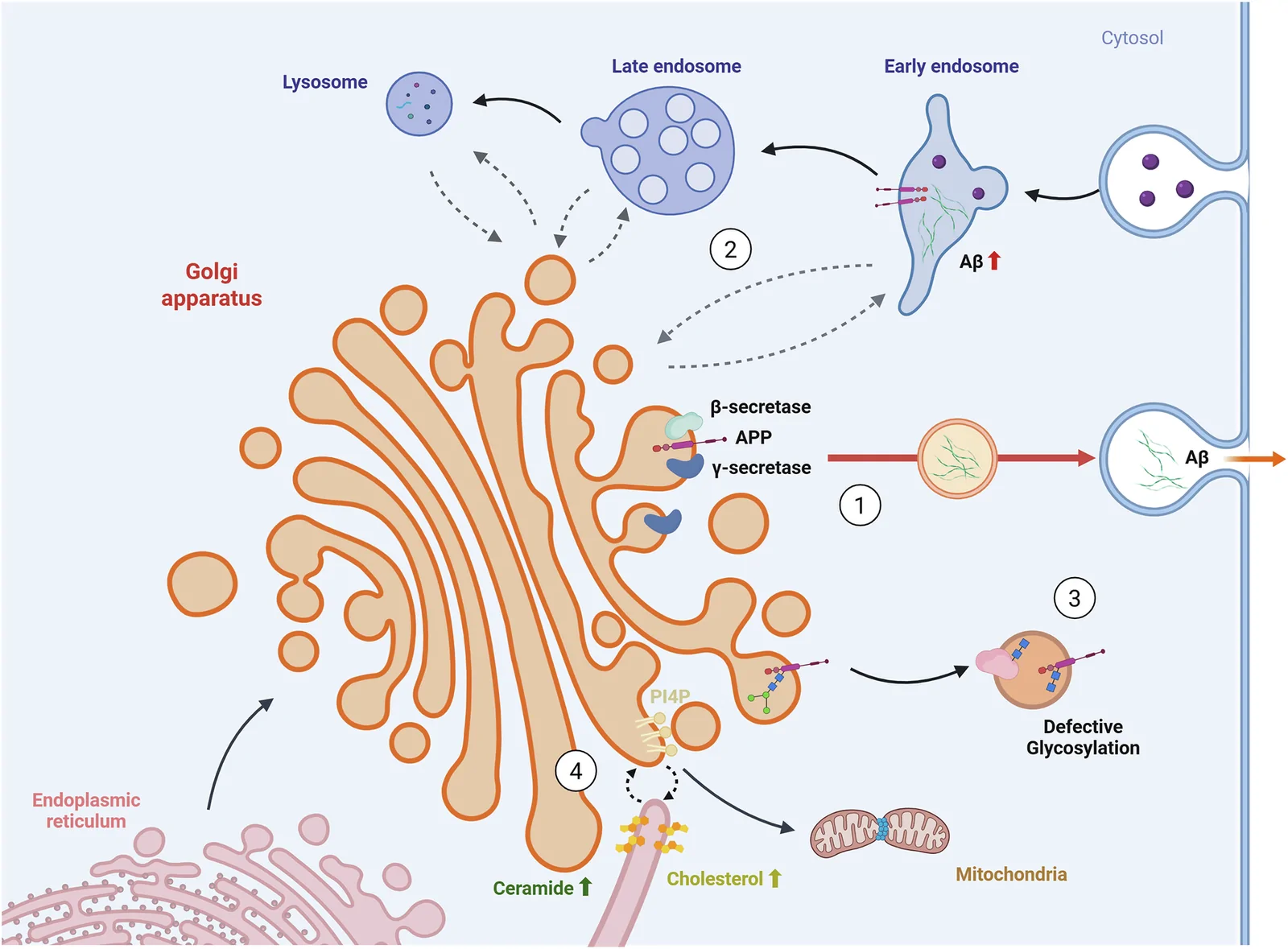
Summary of suggested Golgi–AD pathogenic mechanisms. (1) Disrupted segregation of APP and APP-processing enzymes. (2) Impaired trafficking between Golgi and endolysosomal compartment. (3) Changes in protein modifications. (4) Altered lipid homeostasis.

Golgi dysfunction contributes to AD pathogenesis via various mechanisms. Here we summarize possible Golgi–AD mechanisms into four categories (Fig. 6):

1. Disrupted segregation of APP and APP-processing enzymes
  Disruption of Golgi structure can compromise the trafficking of APP and BACE1 that is normally segregated under physiologic conditions, increasing the possibility of contact between them^115^. Furthermore, TGN is reportedly one of the major sites of Aβ production in several studies^116,117^. The possible mechanisms of abnormal Aβ production in the Golgi have been thoroughly addressed in previous reviews^118,119^. In addition, Golgi fragmentation can accelerate protein trafficking and thereby increase the secretion of molecules^120^, which can also affect the secretion of Aβ.
2. Impaired trafficking between Golgi and endolysosomal compartment
  Endolysosomal dysfunction can increase the production and decrease the clearance of Aβ^59^. Golgi dysfunction might be one of the causes of endolysosomal dysfunction in AD. TGN communicates closely with endolysosomal compartments, exchanging molecules to maintain proper function of the organelles. Genetic risk factors that are involved in endosome–Golgi trafficking strongly imply pathogenic involvement of this process. Reduced APP retrograde trafficking from endosome to TGN results in APP accumulation within the endosomes, promoting amyloidogenic cleavage. Moreover, sorting of lysosomal proteins at Golgi is critical to lysosomal protein degradation. Dysfunctional Golgi would compromise protein trafficking and aggravate lysosomal defects, thereby increasing Aβ production and impairing protein degradation.
3. Changes in protein modifications
  Disruption of trafficking throughout Golgi can lead to failure of glycosylation by Golgi-resident glycosyltransferases, affecting numerous proteins, including matrisome proteins and even APP. APP reportedly has several O-glycosylated residues in AD^121^, and this O-glycosylation of APP affects trafficking and beta-cleavage^122,123^. As Golgi is the primary site of O-glycosylation^69^, defects in O-glycosylation in Golgi would increase Aβ production. Because Golgi is heavily involved in posttranslational protein modifications, especially glycosylation, its defects represent a plausible pathogenic link driving pathogenic protein modifications, which render proteins prone to aggregation or reduce their clearance. Interestingly, the knockdown of Golgi-resident cation channel TMEM87A in astrocytes, the main ECM organizer in the brain, resulted in altered glycosylation profile and impaired hippocampus-dependent memory function^124^, implying that dysregulation of protein modification throughout Golgi can affect cognitive function.
4. Altered lipid homeostasis
  Golgi is implicated in the metabolism of a variety of lipid species. Although Golgi dysfunction can increase ceramide levels as observed in AD, imbalance of lipids conversely can impair Golgi membrane integrity and function, forming a vicious cycle. Furthermore, Golgi-mediated cholesterol dysregulation can directly impair ER and mitochondrial function to accelerate AD pathogenesis^125,126^. In addition, cholesterol dysregulation at Golgi might also affect Aβ production at lipid rafts, where amyloidogenic cleavage happens^127^.

## Therapeutic implications

**Fig. 7:**
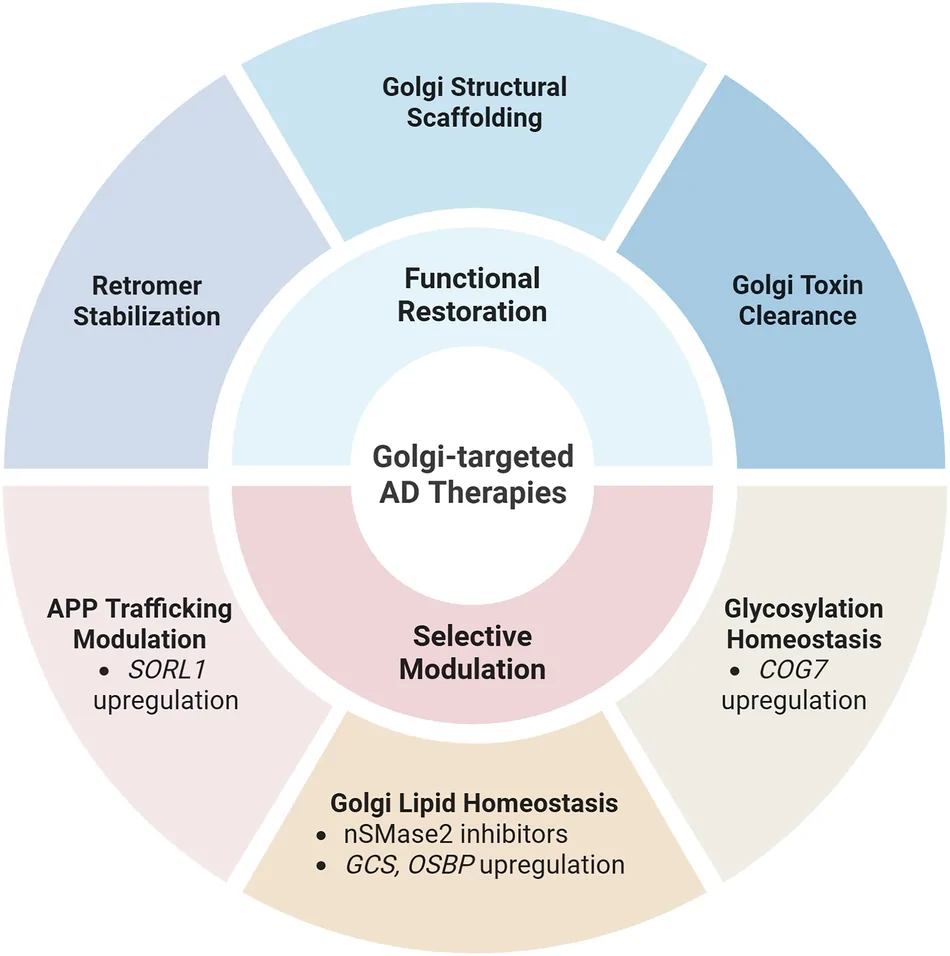
Golgi-targeted therapeutic strategies for AD. Most of the strategies shown are currently conceptual or supported only by in vitro evidence and require further validation, including in vivo studies.

Despite the need for more mechanistic studies, Golgi holds therapeutic potential for treating AD (Fig. 7). Restoring Golgi integrity enables improving multiple aspects of disease pathogenesis simultaneously. Currently, limited studies have shown therapeutic approaches targeting Golgi for AD treatment.

One possible approach is direct enhancement of Golgi structural integrity. Expression of nonphosphorylatable GRASP55/65 reduced Golgi fragmentation and subsequent Aβ production^20^. Because Golgi structure is tightly coupled to its functions, such strategy is expected to produce multidimensional benefits. Therapeutic viability of this approach should be evaluated in other experimental models, especially in vivo. Therapeutic agents that scavenge Golgi-toxic stimuli, such as antioxidants, can also help maintain normal Golgi function. Reinforcing normal Golgi function by expression of Golgi proteins or by administration of Golgi-protecting drugs would not only help cells cope with pathogenic processes but also support overall cellular homeostasis.

Alternative options are specific targeting of Golgi-related factors (Table 1). For instance, nSMase2 inhibitors have been shown to reduce extracellular vesicle secretion, plaque load, and tau spread in AD mouse models^89,90,128^. Whether the drugs are applicable to patients should be further tested in clinical trials. Overexpression of *SORL1* may be a promising approach, as overexpression in vitro resulted in redistribution of APP to Golgi and decreased Aβ production^36^. Targeting other critical homeostatic regulators may also be viable strategies, such as reinforcing glycosylation via *COG7* overexpression or restoring lipid homeostasis via overexpression of *UGCG* (GCS) or *OSBP1*.

**Table 1:** Multiomic evidence of Golgi–AD link and potential therapeutic strategies.

Omics layer	Gene/Protein	Change in AD	Functional consequence	Evidence level	Potential therapeutic strategies	Development stage	Major risks
Genomics	SLC9A7^29^	Risk loci (predicted to increase expression)	Change in luminal pH of TGN	Omics	Not established	-	-
	NSF^43^	Protective loci	Impaired Golgi reassembly and vesicle fusion	Omics	Promoting Golgi reassembly (ex: GRASP55/65 overexpression^20^)	Assessed in vitro	May decrease secretion and glycosylation of diverse proteins
	SORL1^35^	Risk loci (loss of function),↓^129^	Dysregulation of trafficking between TGN and endosomes	In vivo rescue^130,131^	SORL1 overexpression^36^	Assessed in vitro	Cell type-specific effects besides neurons need investigation
	SORCS1^38^	Risk loci,↓^38^		In vitro modeling^39,40^			
	AP4M1, AP4E1^41^	Risk loci (enhancer region)	Dysregulation of anterograde trafficking from TGN to endosomes/lysosomes	In vitro modeling^42^	Not established	-	-
Genomics, proteomics	COG7^47,51^	Risk loci	Aberrant glycosylation	Omics	COG7 overexpression	Conceptual	Needs phenotyping of overexpression models
Transcriptomics	QPCTL^54^	↓	Reduced pyroglutamation, possibly affecting neuronal synapse engulfment	Omics	Not established	-	-
Proteomics	SNX1, SNX32, STX6^51^	Risk loci	Dysregulation of endosome-to-TGN trafficking	Omics	Retromer stabilizing drugs^132^	Preclinical stage	May alter trafficking of diverse proteins
	MAN2A1, MAN2A2^76^	↑ ↑	Increased complex N-glycan processing	Omics	Not established	-	-
	MGAT1, MGAT4C^76^	↑ ↓	Dysregulated complex N-glycan processing	Omics	Not established	-	-
	B3GALT2, B4GALT1, B4GALT4^76^	↓ ↑ ↑	Dysregulated galactosylation	Omics	Not established	-	-
	ST6GAL1, ST6GAL2, ST8SIA2, ST8SIA3, ST8SIA4^76^	↑ ↓ ↓ ↓ ↓	Dysregulated sialylation	Omics	Not established	-	-
Lipidomics (indirect evidence)	GCS (UGCG)	↓^87^	Ceramide accumulation	In vitro modeling^87^	nSMase2 inhibitors^89,90,128^ Ceramide-reducing agents^133^	Preclinical stage	May alter intercellular/intracellular signaling mediated by ceramides
	nSMase2 (SMPD3)	↓^92^	Increased activity leads to ceramide accumulation and extracellular vesicle-mediated pathology propagation	In vivo rescue^90,91^			
	OSBP1	-	Organelle cholesterol imbalance	In vitro modeling^105^	OSBP overexpression^105^	Assessed in vitro	May increase lipogenesis

As most of the proposed strategies are currently conceptual or have only been validated in vitro, thorough validation of their therapeutic potential is needed. When developing such therapeutic strategies, potential side effects and tissue/cell type specificity must be considered. Future mechanistic studies, together with integrative screening methodologies, would facilitate development the of Golgi-targeting treatment strategies for AD.

## Conclusions

Despite the functional importance and interactions with other organelles, Golgi has long been an underappreciated organelle in AD research. Multiple omics studies have revealed that Golgi has strong links to the disease. Owing to rapid technological advances, the research field is continuously discovering novel risk factors and disease mechanisms leveraging large human data. Identification of specific Golgi-related risk factors directly supports its relevance in AD, whereas data from proteomic and lipidomic studies provide further evidence of involvement in various disease-related processes. Dysfunction of Golgi can impair processing, trafficking, and secretion of proteins and lipids, ultimately predisposing the intracellular/extracellular environment to disease pathogenesis. Golgi might be a pivotal point of linking various pathogenic mechanisms.

A critical point to consider is that causality between Golgi dysfunction and AD pathogenesis has not been thoroughly explored. Although several studies have reported potential causal links, they are generally conducted in a limited context (for example, using familial AD model such as APPswe cells). More experimental studies using sporadic AD models, in addition to large-scale real-world human data, would help clarify the causal link and underlying pathogenic mechanism involving Golgi dysfunction. Furthermore, cell type-specific analyses based on high-resolution omics data, such as single-cell transcriptomics, spatial transcriptomics, and spatial proteomics, are needed to delineate specific cell types most vulnerable to Golgi defects and their contribution to pathogenesis.

Another important limitation is that Golgi dysfunction may not selectively affect AD-related processes, but rather broadly influence the trafficking and metabolism of a large number of proteins. Although many studies, including this review, highlight specific Golgi-associated proteins implicated in AD pathogenesis, an overemphasis on individual factors may obscure the global cellular consequences of Golgi disruption. Therefore, Golgi-related findings should be interpreted within a broader context, recognizing that maintaining Golgi integrity is essential for overall cellular homeostasis. In this regard, maintaining ‘healthy’ Golgi would be the key to preserving normal function of cells and preventing progression of AD pathogenesis. Future studies should focus further on developing Golgi-targeted therapeutic strategies based on Golgi-mediated pathogenic mechanisms, with deliberate consideration of potential pleiotropic effects. Multiomic studies would provide valuable insights into evaluating the importance of Golgi in AD and also offer novel therapeutic options to halt disease progression.
